# New OprM structure highlighting the nature of the N-terminal anchor

**DOI:** 10.3389/fmicb.2015.00667

**Published:** 2015-07-01

**Authors:** Laura Monlezun, Gilles Phan, Houssain Benabdelhak, Marie-Bernard Lascombe, Véronique Y. N. Enguéné, Martin Picard, Isabelle Broutin

**Affiliations:** ^1^Laboratoire de Cristallographie et RMN Biologiques, CNRS UMR 8015, Faculté de Pharmacie, Université Paris DescartesParis, France; ^2^Laboratoire d’Imagerie Biomédicale, Sorbonne Universités, Université Pierre et Marie Curie Paris 6, CNRS UMR 7371, INSERM U1146Paris, France

**Keywords:** multidrug resistance, eﬄux pump, membrane protein, post-translational modification, lipoyl, X-ray structure

## Abstract

Among the different mechanisms used by bacteria to resist antibiotics, active eﬄux plays a major role. In Gram-negative bacteria, active eﬄux is carried out by tripartite eﬄux pumps that form a macromolecular assembly spanning both membranes of the cellular wall. At the outer membrane level, a well-conserved outer membrane factor (OMF) protein acts as an exit duct, but its sequence varies greatly among different species. The OMFs share a similar tri-dimensional structure that includes a beta-barrel pore domain that stabilizes the channel within the membrane. In addition, OMFs are often subjected to different N-terminal post-translational modifications (PTMs), such as an acylation with a lipid. The role of additional N-terminal anchors is all the more intriguing since it is not always required among the OMFs family. Understanding this optional PTM could open new research lines in the field of antibiotics resistance. In *Escherichia coli,* it has been shown that CusC is modified with a tri-acylated lipid, whereas TolC does not show any modification. In the case of OprM from *Pseudomonas aeruginosa,* the N-terminal modification remains a matter of debate, therefore, we used several approaches to investigate this issue. As definitive evidence, we present a new X-ray structure at 3.8 Å resolution that was solved in a new space group, making it possible to model the N-terminal residue as a palmitoylated cysteine.

## Introduction

After several decades of continuous antibiotic therapy success, we are now facing the appearance of multi-drug resistant strains and the near absence of new antibiotic family development for more than 10 years (Fischbach and Walsh, 2009; Hede, 2014). These facts highlight the need for new anti-infection strategies (Walsh, 2003; Olivares et al., 2013), although a promising compound isolated from natural soil bacteria that is able to kill Gram-positive pathogens, was recently reported (Ling et al., 2015). Among the most virulent nosocomial pathogens are *Pseudomonas aeruginosa, Escherichia coli, Staphylococcus aureus, Enterococci,* and *Acinetobacter baumannii* (Poole, 2004; Lister et al., 2009; Bereket et al., 2012; Bayram et al., 2013). These strains have developed several resistance strategies including active eﬄux pumps (Cattoir, 2004; Li and Nikaido, 2009; Nikaido, 2009; Nikaido and Pages, 2012). In Gram-negative bacteria eﬄux pumps are multimers of three different proteins that form a long transmembrane scaffold linking the cytoplasm to the outside of the cell (Nikaido and Pages, 2012). These tripartite assemblies are composed of an inner membrane protein from the RND (Resistance Nodulation cell Division) family, which corresponds to the pumping motor that uses the proton-gradient as an energy source; an outer membrane channel from the OMF (Outer Membrane Factor) family; and a periplasmic protein from the MFP (membrane fusion protein) family, anchored to the inner membrane and connecting the other two proteins. In *P. aeruginosa,* up to 12 different pumps have been sequenced (Yen et al., 2002), including OprM_OMF_-MexA_MFP_-MexB_RND_ which is one of the most studied pumps because of its constitutive expression whereas the others appear under antibiotic pressure (Li and Poole, 2001; Nakajima et al., 2002; Schweizer, 2003). This study will focus on the versatile membrane channel OprM, which has the ability to work with at least four different pumps, including OprM-MexAB, OprM-MexXY (Aires et al., 1999; Morita et al., 2012), OprM-MexJK (Chuanchuen et al., 2002), and OprM-MexMN (Mima et al., 2005).

**FIGURE 1:**
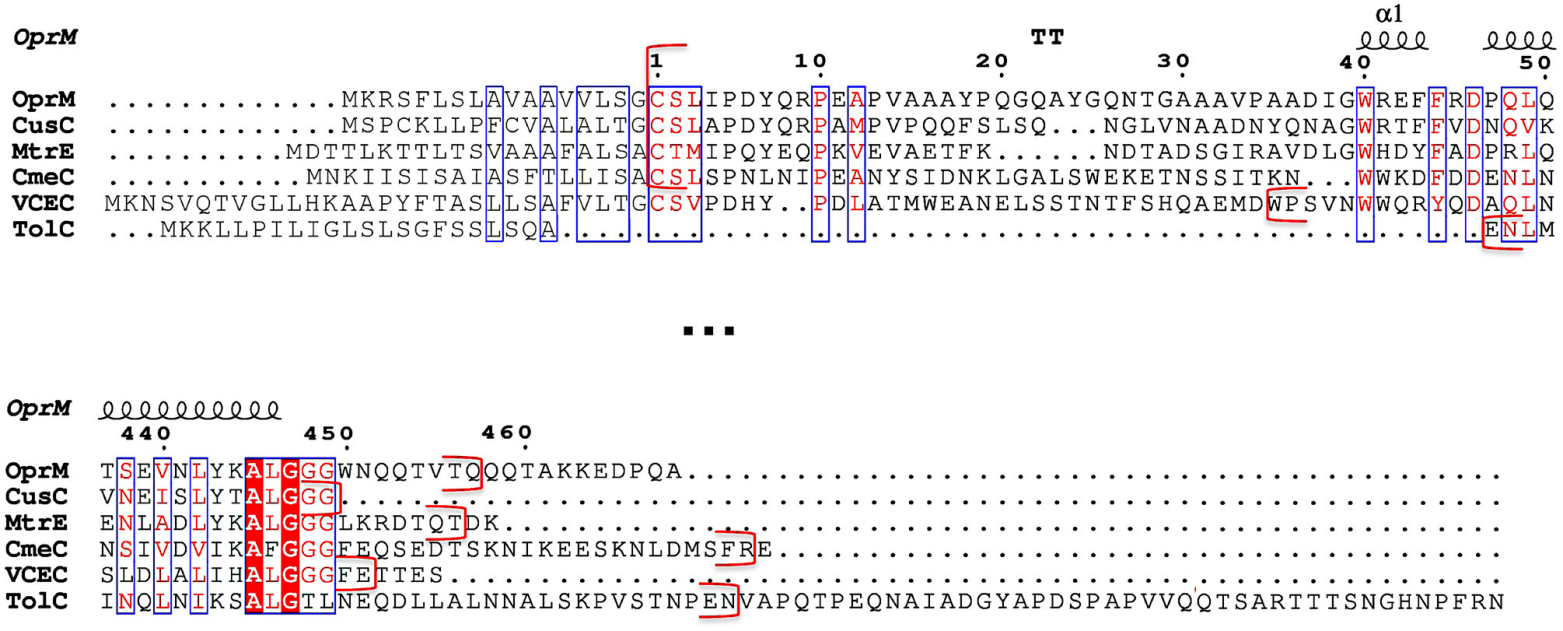
**Sequence alignment of the six outer membrane factor (OMF) proteins with known structures.** Only the N- and C-terminal portions of the alignment (extract from Supplementary Figure S1) corresponding to the most divergent 3D structure regions of these OMF proteins are shown. The numbering corresponds to the OprM sequence after cleavage of the targeting signal presented in grey letters. The secondary structure of OprM is indicated at the top of the aligned sequences. The red brackets indicate the beginning and end of each protein resolved PDB structure.

The X-ray structure of OprM was solved in two different space groups showing its trimeric nature [Protein Data Bank (PDB) code: 1WP1 (Akama et al., 2004); 3D5K (Phan et al., 2010)]. The architecture of OprM is composed of a beta-barrel domain that is ∼40 Å in height spanning the outer membrane and a periplasmic alpha-helical domain that is ∼100 Å in length and bears a central buoy. Most of the structure has been determined with the exception of the eleven C-terminal amino acids, which are not visible in the electronic density maps of both structures. In addition, the structure of the post-translational modification (PTM) that covalently links the Cys-terminal residue to a lipoyl has never been properly characterized at any resolution, despite palmitoylation being suggested some time ago (Nakajima et al., 2000). This lipoyl modification is not commonly shared among OMF family members. For instance, the *E. coli* homolog TolC (PDB code: 1EK9, Koronakis et al., 2000), has an N-terminus that is 44 residues shorter and does not begin with a cysteine. Other OMFs with known structures, such as VceC from *Vibrio cholerae* (PDB code: 1YC9, Federici et al., 2005), CusC of the metal eﬄuent pump from *E. coli* (3PIK, Kulathila et al., 2011; 4K7R, Lei et al., 2014a) and CmeC from *E. coli* (4MT4, Su et al., 2014) begin with residues Cys-Ser (**Figure 1**; Supplementary Figure S1) like OprM. The OMF protein with the most recently solved structure, MtrE (4MT0, Lei et al., 2014b) from *Neisseria gonorrhoeae,* begins with Cys-Thr. The structure of CusC indicates the presence of a di-acylated thiol on the N-terminal cysteine, with or without a supplementary acyl chain on the N-terminal amine (tri-acylation), depending on the protomer. As the CusC sequence is highly similar to OprM, it has been suggested that the latter may be modified in a similar manner, rather than with only one palmitoyl chain attached by a thio-acyl bound to the N-terminal Cys as previously described (Kulathila et al., 2011). Di-acylation is also found in CmeC, but nothing has been found to be added to the N-terminal cysteine of the MtrE structure. In the VceC structure, the protein sequence was cloned without the first 12 amino acids, therefore, the N-terminal cysteine was not present. In the OprM structure solved in the R32 space group (Akama et al., 2004), the three monomers were found to be identical by the threefold crystallographic symmetry axis, and despite a resolution of 2.6 Å there was no evidence of lipidation to the thiol or amine of the N-terminal cysteine. The same observation applies to the OprM structure solved at 2.4 Å of resolution (Phan et al., 2010) in which the P2_1_2_1_2_1_ space group does not stabilize sufficiently the N-terminal modified cysteine.

Consequently, the N-terminal modification of OMFs remains an open question. Post-translational lipidation is particularly essential for the secretion and localization of some membranous proteins, a process involving different biological modifiers such as palmitoyl acyl transferases (Aicart-Ramos et al., 2011), palmitoyl thioesterases, lipoprotein diacylglyceryl transferase or lipoprotein *N*-acyl transferase (Linder and Deschenes, 2007; Kovacs-Simon et al., 2011; Nakayama et al., 2012). These modifying enzymes could potentially lead to new therapeutic targets. In addition, it is not known why some OMF proteins need to undergo PTM in addition to their *trans*-membrane beta-barrel insertion, or what is the nature of this modification. We asked this question using OprM from *P. aeruginosa* with specific N-terminal chemical probes to investigate whether it is actually palmitoylated via a thio-acyl or subjected to other PTMs, and resolved a new OprM structure in a new crystallographic space group.

## Materials and Methods

### Expression and Purification of OprM, MexAp, and MexAnp

The three proteins were produced following the protocol described by Phan et al. (2010) and Ferrandez et al. (2012) with some modifications. The protein genes were inserted into the pBAD33-GFPuv plasmid with a C-terminal 6-histidines tag and the N-terminus extremity being dedicated to the signal peptide. For the non-palmitoylated form of MexA (MexAnp), the signal peptide was deleted from the construct, resulting in a non-membranous protein bearing a free N-terminal cysteine. Wt MexA has the same amino acid sequence as MexAnp, but its starting cysteine is palmitoylated after maturation (MexAp).

The plasmids were transformed into the C43-DE3 *E. coli* strain (Miroux and Walker, 1996). For each protein 6 L of cultures were grown; they were begun at OD_600_ = 0.05 from dilution of an overnight pre-culture at 37°C in LB medium containing 25 μg/ml chloramphenicol, and were then grown at 30°C. Cells were induced at OD_600_ = 0.6–0.8 by the addition of 0.02% L-arabinose and grown for 2 h before centrifugation for 20 min at 9,000 *g*. The cell pellet was resuspended in 45 ml of buffer containing 20 mM Tris-HCl (pH 8.0), 5 mM MgCl_2_, 1 μl/ml cocktail of anti-protease inhibitors set III (Calbiochem) and 50 units of benzonase (Promega).

Cells were broken using a French pressure cell at 69 MPa and then centrifuged twice for 30 min at 8,500 *g* to remove inclusion bodies and unbroken cells. As for OprM, the supernatant was applied to a sucrose step gradient (0.5 and 1.5 M) and then centrifuged for 3 h at 200,000 *g* at 4°C for membrane separation. The pellet, corresponding to the outer membrane fraction, was re-suspended in a solution containing 20 mM Tris-HCl (pH 8.0), 10% glycerol (v/v) and 2% βOG (w/v) (Anatrace) and then stirred overnight at 23°C. The solubilized membrane proteins were recovered by centrifugation for 30 min at 50,000 *g*. For MexAp, the lysis supernatant was directly centrifuged at 100,000 *g* for 1 h at 4°C, and the pellet was resuspended in a solution containing 20 mM Tris-HCl (pH 8.0), 10% glycerol (v/v), 2% βOG (w/v) (Anatrace) 0.2% *N*-lauryl sarkosyl, and 15 mM imidazole, then stirred overnight at 23°C. The solubilized membrane proteins were recovered by centrifugation at 4°C for 1 h at 100,000 *g*. For MexAnp, because it is produced directly in the cytoplasm, there is no need for a detergent solubilization step. After lysis and centrifugation, 15 mM imidazole was added to the supernatant before loading onto the column. For the three proteins, the same protocol was then used. The proteins were loaded onto a Ni-NTA resin column pre-equilibrated with 20 mM Tris-HCl (pH 8.0), 200 mM NaCl, 10% glycerol (v/v), 15 mM imidazole for MexAnp, the same buffer plus 0.9% βOG (w/v) for OprM, and the addition of 0.2% *N*-lauryl sarkosyl for MexAp. After washing of the column, the proteins were eluted in the same respective buffers containing 300 mM imidazole, and then desalted using a PD-10 desalting column (GE) to remove the imidazole. MexAp and MexAnp were concentrated up to 2.5 mg/ml. OprM was concentrated up to 8 mg/ml using the 30-kDa cutoff Amicon system (Millipore).

### Labeling of the N-Terminal Amine

The fluorescent compound 4-chloro-7-nitrobenzofurazan (NBD-Cl) is a fluorogenic reagent that reacts with protein N-terminal amines but not with lysines in the conditions used as their respective pKa largely differ (Ghosh and Whitehouse, 1968; Bernal-Perez et al., 2012). A NBD-Cl stock solution was prepared in DMSO (dimethylsulfoxide) and 6 μM of OprM in 50 mM Hepes buffer (pH 7.5) and 0.05% DDM (w/v) containing 1 mM EDTA was mixed with 0.5 mM of NBD-Cl at 4°C. After 6 h the reaction was stopped by adding SDS-PAGE loading buffer [60 mM Tris-HCl (pH 6.8), 25% glycerol (v/v), 2% SDS (w/v), 0.1% bromophenol blue (w/v)] and the solution was deposited on an SDS gel together with unmarked OprM protein. The gel was analyzed for fluorescence using a UV transilluminator at an emission wavelength of 504 nm. A clear band was visualized for the labeled OprM.

### Labeling of the N-Terminal Cysteine Sulfur

The fluorescent compound MTS-EMCA [*N*-(2-Methanethiosulfonylethyl)-7- methoxycoumarin-4-acetamide, Toronto Research, Chemicals Inc.] is a sulfhydryl active reagent that covalently attaches to the reduced cysteine via a disulfide bond. 25 mM proteins (MexAp, MexAnp, and OprM) were incubated for 2 h with 2 mM MTS-EMCA in 10 mM HEPES pH 7.5, 150 mM NaCl, βOG 1% (w/v), at room temperature away from light. Reactions were stopped by adding an equivalent volume of SDS-PAGE loading buffer. After electrophoresis, gels were visualized with a UV transilluminator at 312 nm before coomassie blue staining. MTS-EMCA fluorescence appeared for the labeled proteins.

### Crystallization and Data Collection

OprM crystals were grown by vapor diffusion using the hanging drop method. Two different crystal forms were obtained, both leading to the recording of a diffraction dataset after 100s of crystal tests on the different beam lines of both SOLEIL and ESRF synchrotrons. One crystal was obtained in a 1 M sodium citrate (pH 5-6) precipitation solution and was found to belong to the P2_1_2_1_2_1_ space group. The structure in this space group has been previously published (Phan et al., 2010). The second crystal was obtained in 100 mM sodium acetate (pH 4.5), 6% PEG 20 000 (w/v), 300 mM ammonium citrate, 25-30% glycerol (v/v), and 0.9% βOG (w/v). These rhombohedral crystals (100 μm × 100 μm × 30 μm) belong to the C2 space group and diffracted to 3.8 Å resolution. A complete dataset was collected on beamline ID29 (ESRF, Grenoble) with an exposure time of 10 s per degree of oscillation. Owing to its low resolution, this data set has been kept for a long time without solving the structure, but the recent question of the N-terminal modification nature prompted us to ultimately solve the OprM structure in the C2 space group.

### Data processing, Model Building, and Refinement

Reflections were integrated, scaled and reduced using the programs XDS (Kabsch, 1993) and TRUNCATE from the CCP4 suite (1994). Data collection statistics are summarized in **Table 1**.

**Table 1:** Crystallographic data and refinement statistics.

Data collection	
Beamline	ESRF ID29
X-ray wavelength (Å)	1.0052
Crystal – detector distance (mm)	400
Space group	C2
Cell dimensions	
*a, b, c* (Å)	152.6, 87.9, 355.9
α, β, γ(°)	90, 98.9, 90
Matthews coefficient (Å^3^/Da)	3.80
Solvent content (%)	67.7
Resolution (Å)^a^	87.9 - 3.8 (3.9–3.8)
Number of reflections	131 301
Number of unique reflections	40 566 (3571)
*R*_merge_ *(%)*^a,b^	12.2 (31.2)
Completeness (%)^a^	87.7 (77.9)
Redundancy^a^	3.2 (3.3)
*I/sigma*^a^	8.5 (3.3)
**Refinement**	
*R*_work_*/R*_free_ *(%)*^c,d^	29.7/34.6
Number of residues	2 741
Number of solvent molecules	0
Rmsd from ideal values	
Bonds (Å)	0.010
Angles (°)	1.400
Mean B-factor (Å)^2^	94.2

^a^Values in brackets correspond to the highest resolution shell.

^b^R_*merge*_ = Σ_*h*_Σ_*i*_∣I(h)_*i*_ - <I(h)>∣/Σ_*h*_Σ_*i*_ <I(h)> where I(h) is the observed intensity.

^c^R_*work*_ = S_*hkl*_ || F_*obs*_| - | F_*calc*_|| /S_*hkl*_ | F_*obs*_| .

^d^R_*free*_ was calculated for 7% of reflections randomly excluded from the refinement.

We have solved the C2 structure of OprM by molecular replacement with PHASER (McCoy et al., 2007) in automatic mode using our previously solved structure (PDB code 3D5K, Phan et al., 2010) as a model. Refinements were conducted using Phenix (Adams et al., 2002) and the protein was rebuilt with COOT (Emsley and Cowtan, 2004). The Bfactors were refined by groups. These groups were determined after a refinement step using the two Bfactors per residues option, but the resulting structure was not kept for the continuation. The TLS option was not used.

The last 19 C-terminal residues could not be assigned, probably due to the large flexibility of this region. The validity of our model was checked using MolProbity (Davis et al., 2007) and the polygon tool (Urzhumtseva et al., 2009) from Phenix (see Supplementary Figure S3). The OprM structure model of the C2 crystal was deposited in the PDB (4Y1K).

Figures were created with Pymol (DeLano, 2002).

### Sequence Alignment

Sequence alignment of the different OMF proteins whose structures were deposited in the PDB was performed using the program MUSCLE (**MU**ltiple **S**equence **C**omparison by **L**og- **E**xpectation)^1^. The alignment was submitted to ESPript 3.0 (Robert and Gouet, 2014) for customization.

## Results

### Chemical Analysis of the Lipoyl Position

**FIGURE 2:**
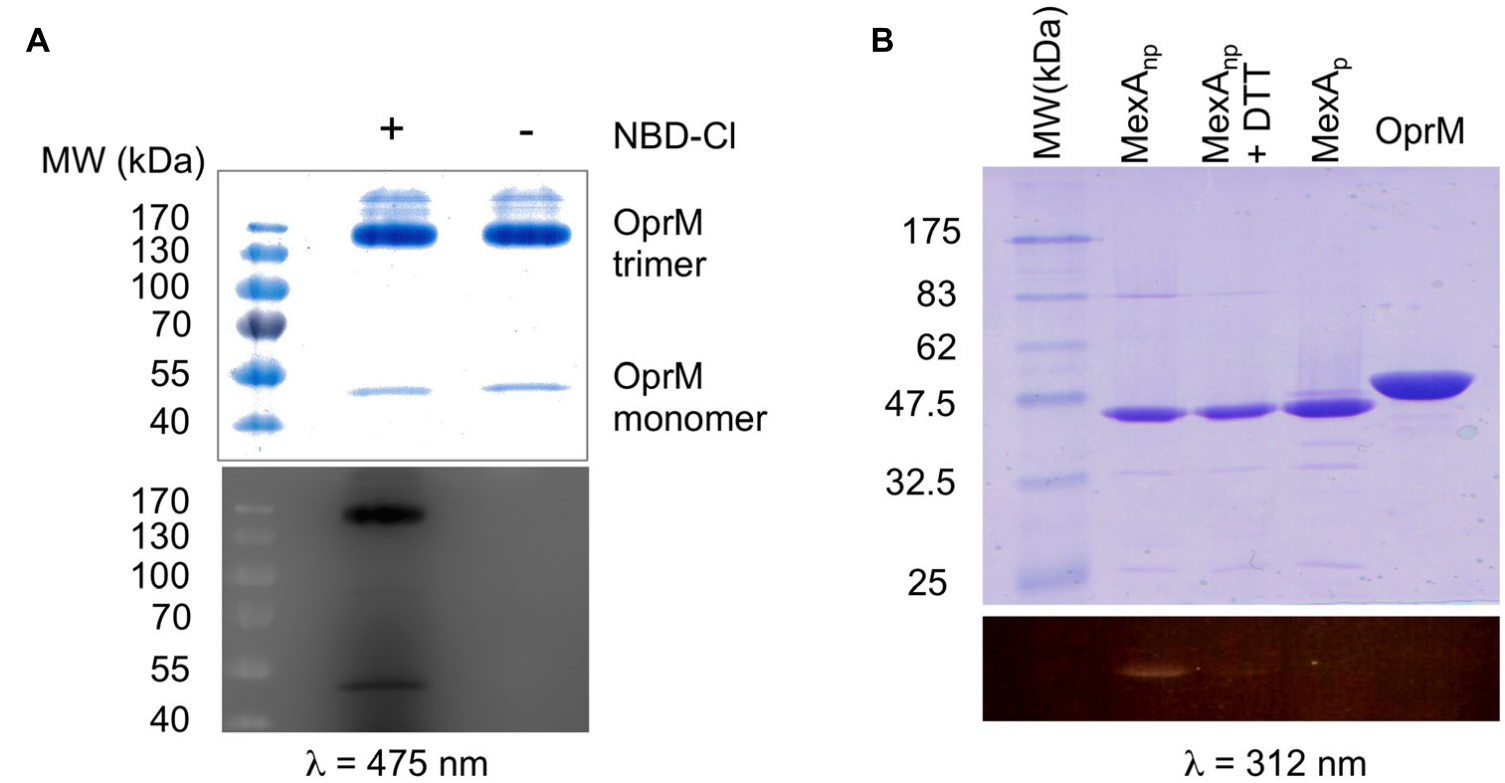
**Chemical analysis of the N-terminal cysteine of OprM. (A)** SDS-PAGE of the NBD-Cl (4-Chloro-7-nitrobenzofurazan)-labeled protein: non-boiled OprM incubated with (+) or without (-) 0.5 mM NBD-Cl. Note that most of the non-boiled OprM remained as a trimer. **(B)** SDS-PAGE of MTS-EMCA [*N*-(2-Methanethiosulfonylethyl)-7-methoxycoumarin-4-acetamide]-labeled proteins: MexAnp with a free N-terminal cysteine (lane 1), MexAnp plus 5mM DTT (lane 2), MexAp with a palmitoylated N-terminal cysteine (lane 3), and boiled OprM (lane 4). A coomassie blue coloration shows all of the purified proteins. Fluorescence visualization of NBD-Cl (λ = 475 nm) that shows that the N-terminal amine of OprM is available **(A)** whereas visualization of MTS-EMCA (λ = 312 nm) shows that the N-terminal thiol of OprM is not available for labeling **(B)** and is thus occupied by the palmitoyl moiety. Only MexAnp with a free N-terminal thiol is available for MTS-EMCA labeling **(B)**.

Among the different PTMs that can occur on an N-terminal cysteine (Chalker et al., 2009) *N*- or *S*-palmitoylation or acetylation are readily observed (Resh, 1999; Tooley and Schaner Tooley, 2014). As these different modifications are regulated by specific transferases, it is important to characterize the exact nature of OprM PTM. Thus, two questions needed to be clarified: what is the chemical nature of the modification, and which group of the amino acid is modified? To address these two questions, two different types of chemical labeling were performed on purified OprM. To analyze the occupancy of the N-terminal amine, this protein was labeled with the fluorogenic molecule 4-chloro-7-nitrobenzofurazan (NBD-Cl) at neutral pH. This molecule has been proven to be a specific probe for the N-terminal amine only (Bernal-Perez et al., 2012). After incubation with NBD-Cl, the protein was analyzed on an SDS gel, revealing a bright fluorescent band when exposed at 475 nm (**Figure 2A**), thus showing that the N-terminal amine of OprM is actually accessible.

Following the same approach, a different probe was used to verify the occupancy of the sulfhydryl group of the cysteine. The fluorescent compound MTS-EMCA, which specifically links to this group, was used to label OprM. As a control we also analyzed MexAp (wt palmitylated MexA), MexAnp (a mutated form of MexA lacking the signal peptide and bearing a free N-terminal cysteine) and MexAnp in the presence of DTT to reverse thiol acylation. After migration on an SDS gel, UV exposure of the gel at 312 nm (**Figure 2B**, lower panel) revealed a bright band for MexAnp and a faint band for MexAnp with DTT but no signal for OprM, demonstrating that the N-terminal cysteine of OprM is occupied on its thiol by lipoyl modification. Therefore, it can be concluded that the lipoyl modification is anchored exclusively to the sulfhydryl group of OprM.

As a third approach, measuring the precise protein mass has also been considered. Indeed, in our case, one needs to distinguish between a palmitoyl (chemical composition C_16_H_32_O_2_ resulting in a mass of 256 Da) and a tri- or di-acyl that can adopt a variable length. As an example, in the CusC structure can be found a tri-acyl composed of C_19_O_5_H_33_ (PDB code: 3PIK) resulting in a mass of 341 Da, or a di-acyl (PDB code: 4K7R) of chemical composition C_14_O_4_H_25,_ resulting in a mass of 257 Da, which is close to the mass of a palmitoyl. This result illustrates how difficult it is to obtain an answer using this technique as several combinations result in the same mass and it is necessary to measure mass with a precision as high as one Dalton, which is far to be routinely achieved to date with membrane proteins of that size. Attempts to address this question in the proteolyzed protein using the electrospray and MALDI techniques have been unsuccessful because the N-terminus peptide was not detected despite the use of different protease enzymes, and even thought 90% of the OprM sequence was covered by the analysis (data not shown).

### OprM Crystal Structure in the C2 Space Group and Comparison with the Structure Solved in P2_1_2_1_2_1_

As the question about the nature of the modification remained unanswered, it has been envisaged to refine the OprM crystallographic structure in a different space group, as different crystal packing could stabilize the N-terminus and eventually reveal the complete structure of the added lipid. We previously solved the structure of OprM at 2.4 Å resolution in the P2_1_2_1_2_1_ space group (Phan et al., 2010) but this structure showed only the beginning of the N-terminal lipoyl. We previously generated several OprM datasets in the C2 space group, but they were set aside without solving their structures owing to their lower resolution. To investigate the lipoyl structure, we decided to solve the structure of the best diffracting dataset limited to 3.8 Å resolution.

**FIGURE 3:**
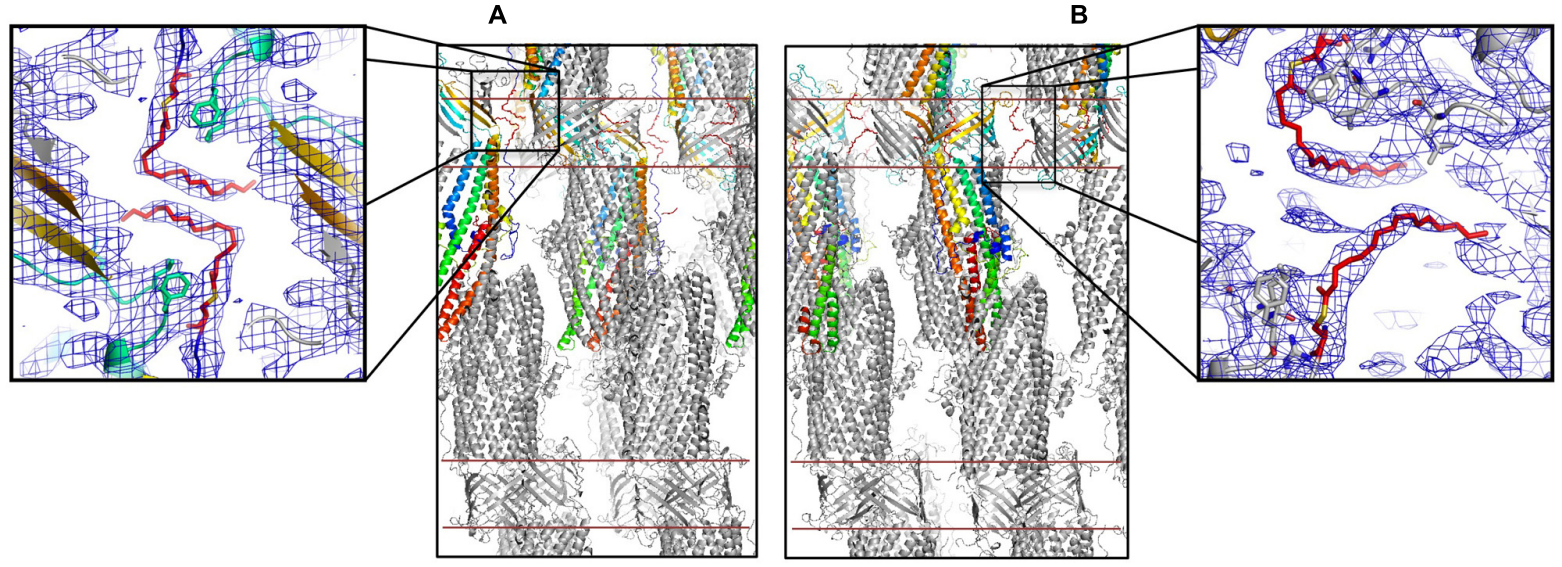
**Palmitoyl modified N-terminal cysteine of OprM.** Packing of the C2 crystal structure of OprM showing the interactions around the palmitate from monomers A **(A)** and B and C **(B)**. The red lines indicate an artificial membrane. Enlargements show the respective electron densities of the palmitoyl modified cysteines (contour at 1 and 0.8 sigma, respectively).

**FIGURE 4:**
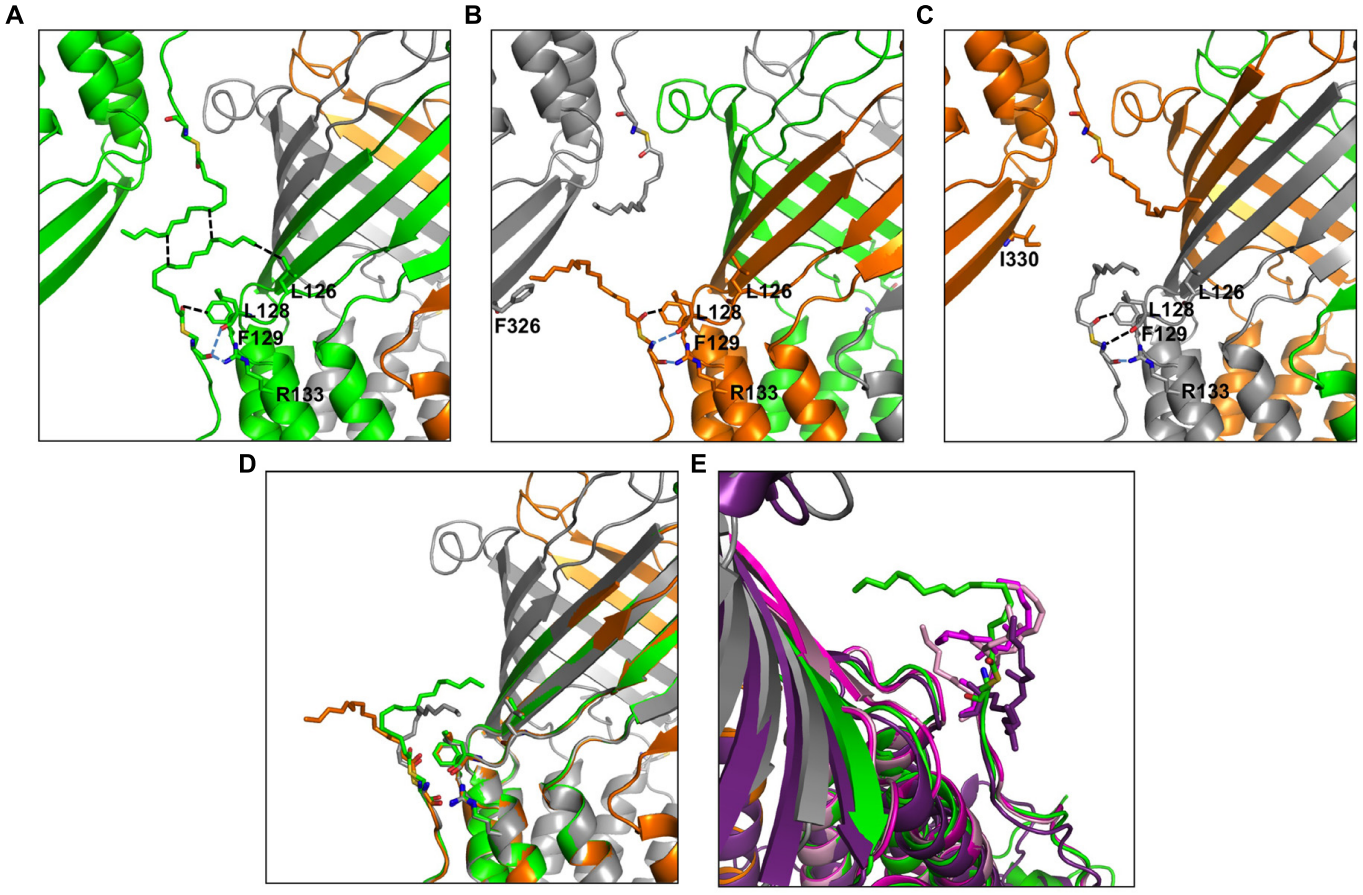
**Environment comparison between palmitates from the three different monomers in the C2 space group. (A–C)** neighboring of the palmitates A, B, and C, respectively. **(D)** Superposition of the three monomers showing different orientations of the palmitate. **(E)** The lipoyl modifications of the CmeC structure (4MT4 in violet) and the two CusC structures (3PIK in light pink, 4K7R in magenta) are superposed on the palmitate from monomer A for length comparison. Monomer A is shown in green, monomer B is shown in orange, monomer C is shown in gray. The ABC trimer from the asymmetric unit together with the closest monomer from packing is represented in each view. The residues in contact with the palmitates are represented as sticks, and the closest contacts are indicated by dotted lines in black for van der Waals and blue for hydrogen bonds.

The here-solved structure comprises two trimers in the asymmetric unit with the second trimer being poorly defined. The crystal packing of our C2 OprM structure is slightly different from the P2_1_2_1_2_1_ structure (see Supplementary Figure S2) but they share common type I crystal packing in which the homo-trimer channels interact in a head-to-head manner through the hydrophobic beta-barrel domains mimicking a lipid bilayer plane. Despite its higher resolution (2.4 Å), the crystal structure of OprM in the P2_1_2_1_2_1_ space group does not reveal the entire palmitoyl moiety because all the three N-termini are oriented toward the solvent and this results in high thermal motions. For two monomers, the closest amino acid is located more than 10 Å away from the expected palmitate main chain, and for the third, only the cysteine portion is clearly constrained, but this is not sufficient to build in the palmitoyl tail. In contrast, when stabilized by a more packed environment around the N-terminus, the entire fatty acid chain appears in the density map of the C2 form where two different orientations are observed (**Figure 3**). This is the sole, but crucial, advantage of this structure, because the crystal packing of OprM is equivalent to that previously published in the R32 space group (Akama et al., 2004) with the exception that the three monomers are not linked by crystallographic symmetries. Attempts were made to highlight some eventual local differences between the monomers even if at low resolution. Superposition of the six different monomers from the C2 asymmetric unit onto monomer A demonstrates a mean Cα-atom RMSD of 0.2 Å for monomers B and C, and 0.58 Å for monomers D, E, and F. These monomer superposition values have to be compared to those obtained for the two other structures of OprM in different space groups (a mean RMSD of 0.50 Å for 440 Cα atoms in both cases) and that of the other OMF solved structures (1.87 Å for CmeC for 377 Cα atoms, 1.28 Å for CusC on 381 Cα atoms, 1.79 Å for VceC on 347 Cα atoms, 1.32 Å for MtrE on 383 Cα atoms, and 2.47 Å on 321 Cα atoms for the closest TolC structure [PDB code: 2VDE (Bavro et al., 2008)]. Although no striking differences among the alternative crystal structures of OprM monomers were revealed, this analysis highlights the highly conserved folding within the OMF family with the most divergent being TolC in accordance with the sequence alignment (Supplementary Figure S1). Thus it appears that the only reason OprM would crystallize in either the R32 or C2 space groups is that the N-terminus lipoyl adopts a different conformation within the three monomers. To understand the different orientations of the palmitoyl, we compared their respective environments (**Figure 4**). For the three N-termini, the main chain is stabilized by hydrogen bonds with R133 and the carboxyl group of L128. In each case, the palmitoyl tail then turns around two hydrophobic residues, L128 and F129. The palmitoyl from the B monomer (subsequently called Palm-B) is located near the Palm-C of a symmetrical molecule but not close enough to form van der Waals contact because the distance between them is greater than 7 Å. Interestingly, Palm-A instead makes short van der Waals contacts with its own symmetric structure, justifying the quality of the electron density map for this region (see **Figure 3**) even at low resolution. The quality of the electron density for this particular monomer makes us confident about the exact nature of the fatty acid modification of OprM. As a final control the N-terminal PTMs that were generated in other OMF protein structures, namely CusC and CmeC (**Figure 4E**) were superimposed on our structure and these demonstrate a longer acyl chain for OprM, supporting its palmitoyl nature.

## Discussion

Post-translational modifications (PTMs) play an important role in cell life as they govern most signaling events. Among the different PTMs, lipidation ranks as the second most common modification after phosphorylation^2^ (dbPTM – database of protein PTMs; Beltrao et al., 2013), anchoring proteins to the membrane and stabilizing their interactions with the lipid bilayer.

It is not well understood why proteins that are embedded within cellular membranes via a large hydrophobic structural domain need supplementary PTMs such as the N-terminal lipidation of OMF proteins. Akama et al. (2004) suggested that these proteins first have to be anchored to the membrane by an N-terminal lipid so that the insertion of their large hydrophobic domain may be triggered. This later step is critical for the correct folding of OMFs. This hypothesis has been reinforced by structural determination of two CusC mutants for which the signal peptide has been conserved but the first cysteine residue after processing is replaced with a serine (C1S-CusC) or is deleted (ΔC1-CusC), (Lei et al., 2014a). The structures result in a random folding of the beta-barrel domain of both mutants together with inappropriate opening of the periplasmic helices. These structures support the essential role of OMF N-terminal lipidation in the membrane insertion mechanism; moreover, they also highlight the possible involvement of membrane-interacting components in the OMF opening process and consequently the eﬄux-pump function. In addition to this function, other functions have been attributed to lipid PTMs. It has been shown that the size of the hydrophobic regions of membrane proteins does not necessarily match the thickness of the cellular membrane (Mitra et al., 2004). Additional hydrophobic elements could then help membrane proteins to fit into membranes of variable thickness, which would explain why some proteins require the addition of 16 carbons, whereas others require only fourteen, depending on the transmembrane domain shape and size.

Regarding OprM, several constructs were previously designed for N-terminal labeling, membrane targeting and antibiotic response experiments (Nakajima et al., 2000) and demonstrated that only when residue 18 is a cysteine is the protein labeled by radioactive palmitate and targeted to the outer membrane. Nevertheless, the three tested OprM mutants (C18G, C18F, C18W) were functional, although none were properly targeted to the outer membrane.

Concerning MexA, the MFP component from the OprM-MexAB eﬄux pump that is also palmitylated at its N-terminus and attached to the inner membrane, when this PTM is missing MexA becomes unable to interact with OprM as highlighted by *in vitro* blue native gel experiments (Ferrandez et al., 2012).

All of these data highlight interest in the study of these lipoproteins and the protein partners involved in their modification. To gain insight into these lipoproteins, it seemed important to identify which modification occurs on OprM. To that end, we analyzed the chemical accessibility of the N-terminal cysteine thiol and amine using different fluorescent probes, which revealed that only the thiol was occupied. In addition, the nature of the attached lipoyl was proven to be a palmitoyl by our determination of the structure of OprM in a new space group that trapped the lipoyl chain at the interface of one monomer with its own symmetric structure.

**FIGURE 5:**
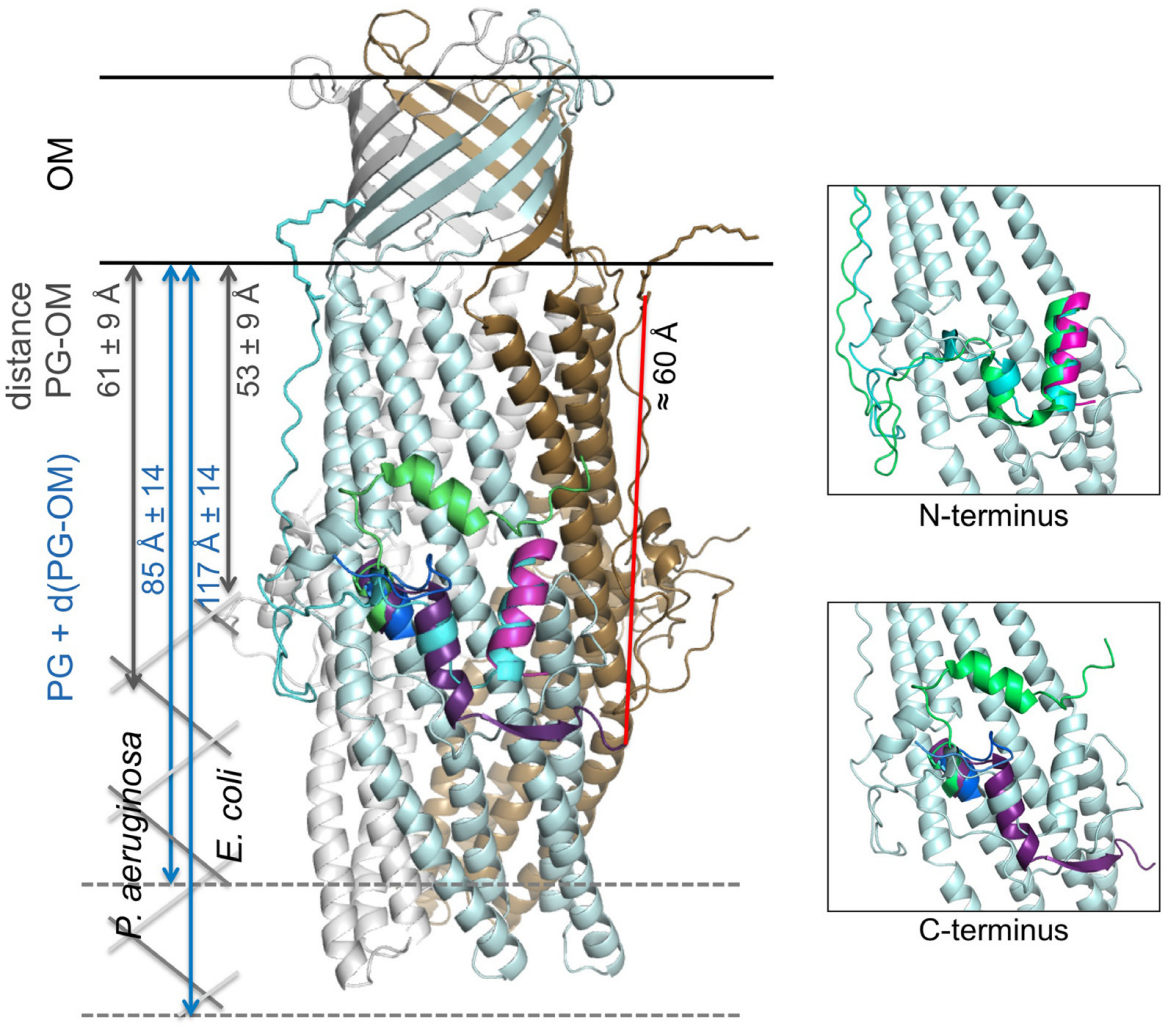
**Cartoon representation of the C2 OprM structure with the superposition of the N- and C-terminal regions of OprM, TolC and CmeC.** The OprM trimer is shown in light blue, gray, and brown. The N-terminal region of OprM is shown in cyan, and its C-terminus is dark blue. The N- and C-termini of TolC are magenta and violet, respectively. Only the C-terminus of CmeC is presented (green) as the N-terminus is similar to that of OprM. The distance between the C-terminal residue present in the TolC structure and the closest N-terminal cysteine from a monomer in the OprM trimer is indicated (red). For comparison, a schematic drawing of the peptidoglycan (PG) has been added at scale for both *Pseudomonas aeruginosa* and *Escherichia coli* (dimension data are from Matias et al., 2003). Two close-up views of the buoy region with only one OprM monomer are presented for clarity. They correspond to the highlighting of the superposition of the N- and C-termini, respectively.

We now know the nature of OprM N-terminal PTM, and that other OMFs such as CusC and CmeC from different bacterial strains, have similar but different N-terminal modifications. Nevertheless, if anchorage was important for the function of this class of proteins, why would the analog TolC not undergo PTMs? The sequences of OprM and TolC were submitted to the prediction of palmitoylation site^3^, which confirmed that C18 was a palmitoylation target in OprM and that there was no such site in the TolC sequence. When comparing the structure of TolC with that of OprM, CusC, and CmeC (**Figure 5**), it appears that only TolC is different at the N-terminus. In particular, TolC is 44 residues shorter in sequence than OprM (**Figure 1**; Supplementary Figure S1), meaning that the TolC structure starts at the buoy level. Nevertheless, it should be noted that although it possesses a shortened N-terminus, TolC has a longer C-terminal tail (**Figure 1**; Supplementary Figure S1), which is not present in its solved structure. Even without knowing anything about the role of this long C-terminal region, it can be hypothesized that it could lead to an additional interaction with the membrane. This hypothesis is supported by the fact that the C-terminal structure of CmeC (an OMF protein with the longest C-terminal sequence of those known) is oriented toward the outer membrane, and the C-terminal alpha-helix of TolC is structurally equivalent to the N-terminal alpha-helix of the other OMFs according to structure superposition (**Figure 5**). In addition, the distance between the last visible C-terminal amino acid of the TolC structure and the membrane proximal N-terminus of the superposed OprM monomer is approximately 60 Å, a distance that could be covered by a 42 residue-long helix, which corresponds to the number of residues missing at the C-terminus of the TolC construct.

The fact that TolC can be properly inserted in the membrane despite its particularity, the absence of lipid anchor, can be due to the differences in the peptidoglycan (PG) structure between Gram-negative bacteria. The NMR structure of a 2 kDa synthetic fragment composed of NAG–NAM (pentapeptides; Meroueh et al., 2006) allowed to visualized TolC as completely embedded in the PG, its periplasmic alpha-helical domain just flushing the limit of the PG. Measurements of the cell wall dimensions of both *E. coli* and *P. aeruginosa* were performed by cryo-transmission electron microscopy (Matias et al., 2003), revealing large size differences between the different cell wall constituents, particularly the PG. The empty space between the PG and the outer-membrane d(PG-OM), and the total thickness, PG + d(PG-OM), were estimated to be 53 ± 9 and 61 ± 9 Å for d(PG-OM), and 117 ± 14 and 85 ± 14 Å for PG + d(PG-OM) in *E. coli* and *P. aeruginosa,* respectively (see **Figure 5**). Consequently, TolC is more likely to be tightly embedded in the membrane barrier than OprM. Nevertheless, no published experiments can support these hypotheses to date.

## Conclusion

In this study, we have shown that OprM is palmitylated at its N-terminal cysteine by thio-palmitoylation. It is now necessary to search for the different proteins involved in this acylation, lipid-transferases, and signal peptidases. These lipoprotein modifiers could represent new interesting targets for the fight against antibiotic resistance.

## Author Contributions

Conceived and designed the experiments: GP, HB, and IB. Performed the experiments: LM, GP, HB, M-BL, YN, and IB. Analyzed the data: LM, GP, HB, M-BL, YN, MP, and IB. Wrote the paper: GP, MP, and IB.

## Conflict of Interest Statement

The authors declare that the research was conducted in the absence of any commercial or financial relationships that could be construed as a potential conflict of interest.

## Footnotes

1 http://www.ebi.ac.uk/Tools/msa/muscle/

2 http://dbptm.mbc.nctu.edu.tw

3 http://csspalm.biocuckoo.org/

## Abbreviations

ßOG: beta-octyl glucopyranoside
DDM: dodecyl maltoside
MexAp: MexA palmitylated
MexAnp: MexA non palmitylated
PTM: posttranslational modification

## References

[B1] Adams P. D. Grosse-Kunstleve R. W. Hung L. W. Ioerger T. R. McCoy A. J. Moriarty N. W. (2002). PHENIX: building new software for automated crystallographic structure determination. *Acta Crystallogr. D Biol. Crystallogr.* 58 1948–1954. 10.1107/S0907444902016657 12393927

[B2] Aicart-Ramos C. Valero R. A. Rodriguez-Crespo I. (2011). Protein palmitoylation and subcellular trafficking. *Biochim. Biophys. Acta* 1808 2981–2994. 10.1016/j.bbamem.2011.07.009 21819967

[B3] Aires J. R. Kohler T. Nikaido H. Plesiat P. (1999). Involvement of an active eﬄux system in the natural resistance of *Pseudomonas aeruginosa* to aminoglycosides. *Antimicrob. Agents Chemother.* 43 2624–2628. 10543738 10.1128/aac.43.11.2624 PMC89534

[B4] Akama H. Kanemaki M. Yoshimura M. Tsukihara T. Kashiwagi T. Yoneyama H. (2004). Crystal structure of the drug discharge outer membrane protein, OprM, of *Pseudomonas aeruginosa*: dual modes of membrane anchoring and occluded cavity end. *J. Biol. Chem.* 279 52816–52819. 10.1074/jbc.C400445200 15507433

[B5] Bavro V. N. Pietras Z. Furnham N. Perez-Cano L. Fernandez-Recio J. Pei X. Y. (2008). Assembly and channel opening in a bacterial drug eﬄux machine. *Mol. Cell* 30 114–121. 10.1016/j.molcel.2008.02.015 18406332 PMC2292822

[B6] Bayram Y. Parlak M. Aypak C. Bayram I. (2013). Three-year review of bacteriological profile and antibiogram of burn wound isolates in Van, Turkey. *Int. J. Med. Sci.* 10 19–23. 10.7150/ijms.4723 23289001 PMC3534873

[B7] Beltrao P. Bork P. Krogan N. J. van Noort V. (2013). Evolution and functional cross-talk of protein post-translational modifications. *Mol. Syst. Biol.* 9 714 10.1002/msb.201304521 PMC4019982 24366814

[B8] Bereket W. Hemalatha K. Getenet B. Wondwossen T. Solomon A. Zeynudin A. (2012). Update on bacterial nosocomial infections. *Eur. Rev. Med. Pharmacol. Sci.* 16 1039–1044. 22913154

[B9] Bernal-Perez L. F. Prokai L. Ryu Y. (2012). Selective N-terminal fluorescent labeling of proteins using 4-chloro-7-nitrobenzofurazan: a method to distinguish protein N-terminal acetylation. *Anal. Biochem.* 428 13–15. 10.1016/j.ab.2012.05.026 22677627

[B10] Cattoir V. (2004). [Eﬄux-mediated antibiotics resistance in bacteria]. *Pathol. Biol.* 52 607–616. 10.1016/j.patbio.2004.09.001 15596311

[B11] CCP4 suite. (1994). The CCP4 suite: programs for protein crystallography. *Acta Crystallogr. D Biol. Crystallogr.* 50 760–763. 10.1107/S0907444994003112 15299374

[B12] Chalker J. M. Bernardes G. J. Lin Y. A. Davis B. G. (2009). Chemical modification of proteins at cysteine: opportunities in chemistry and biology. *Chem. Asian J.* 4 630–640. 10.1002/asia.200800427 19235822

[B13] Chuanchuen R. Narasaki C. T. Schweizer H. P. (2002). The MexJK eﬄux pump of *Pseudomonas aeruginosa* requires OprM for antibiotic eﬄux but not for eﬄux of triclosan. *J. Bacteriol.* 184 5036–5044. 10.1128/JB.184.18.5036-5044.2002 12193619 PMC135324

[B14] Davis I. W. Leaver-Fay A. Chen V. B. Block J. N. Kapral G. J. Wang X. (2007). MolProbity: all-atom contacts and structure validation for proteins and nucleic acids. *Nucleic Acids Res.* 35 W375–W383. 10.1093/nar/gkm216 17452350 PMC1933162

[B15] DeLano W. L. (2002). *The PyMOL Molecular Graphics System*. Available at: http://pymol.sourceforge.net/

[B16] Emsley P. Cowtan K. (2004). Coot: model-building tools for molecular graphics. *Acta Crystallogr. D Biol. Crystallogr.* 60 2126–2132. 10.1107/S0907444904019158 15572765

[B17] Federici L. Du D. Walas F. Matsumura H. Fernandez-Recio J. McKeegan K. S. (2005). The crystal structure of the outer membrane protein VceC from the bacterial pathogen *Vibrio cholerae* at 1.8 A resolution. *J. Biol. Chem.* 280 15307–15314. 10.1074/jbc.M500401200 15684414

[B18] Ferrandez Y. Monlezun L. Phan G. Benabdelhak H. Benas P. Ulryck N. (2012). Stoichiometry of the MexA-OprM binding, as investigated by blue native gel electrophoresis. *Electrophoresis* 33 1282–1287. 10.1002/elps.201100541 22589107

[B19] Fischbach M. A. Walsh C. T. (2009). Antibiotics for emerging pathogens. *Science* 325 1089–1093. 10.1126/science.1176667 19713519 PMC2802854

[B20] Ghosh P. B. Whitehouse M. W. (1968). 7-chloro-4-nitrobenzo-2-oxa-1,3-diazole: a new fluorigenic reagent for amino acids and other amines. *Biochem. J.* 108 155–156. 5657448 10.1042/bj1080155 PMC1198782

[B21] Hede K. (2014). Antibiotic resistance: an infectious arms race. *Nature* 509 S2–S3. 10.1038/509S2a 24784426

[B22] Kabsch W. (1993). Automatic porocessing of rotation diffraction data from crystals of initially unknown symmetry and cell constants. *J. Appl. Crystallogr.* 26 795–800. 10.1107/S0021889893005588

[B23] Koronakis V. Sharff A. Koronakis E. Luisi B. Hughes C. (2000). Crystal structure of the bacterial membrane protein TolC central to multidrug eﬄux and protein export. *Nature* 405 914–919. 10.1038/35016007 10879525

[B24] Kovacs-Simon A. Titball R. W. Michell S. L. (2011). Lipoproteins of bacterial pathogens. *Infect. Immun.* 79 548–561. 10.1128/IAI.00682-10 20974828 PMC3028857

[B25] Kulathila R. Indic M. van den Berg B. (2011). Crystal structure of *Escherichia coli* CusC, the outer membrane component of a heavy metal eﬄux pump. *PLoS ONE* 6:e15610 10.1371/journal.pone.0015610 PMC3017539 21249122

[B26] Lei H. T. Bolla J. R. Bishop N. R. Su C. C. Yu E. W. (2014a). Crystal structures of CusC review conformational changes accompanying folding and transmembrane channel formation. *J. Mol. Biol.* 426 403–411. 10.1016/j.jmb.2013.09.042 24099674 PMC4800009

[B27] Lei H. T. Chou T. H. Su C. C. Bolla J. R. Kumar N. Radhakrishnan A. (2014b). Crystal structure of the open state of the *Neisseria gonorrhoeae* MtrE outer membrane channel. *PLoS ONE* 9:e97475 10.1371/journal.pone.0097475 PMC4046963 24901251

[B28] Li X. Z. Nikaido H. (2009). Eﬄux-mediated drug resistance in bacteria: an update. *Drugs* 69 1555–1623. 10.2165/11317030-000000000-00000 19678712 PMC2847397

[B29] Li X. Z. Poole K. (2001). Mutational analysis of the OprM outer membrane component of the MexA-MexB-OprM multidrug eﬄux system of *Pseudomonas aeruginosa*. *J. Bacteriol.* 183 12–27. 10.1128/JB.183.1.12-27.2001 11114896 PMC94845

[B30] Linder M. E. Deschenes R. J. (2007). Palmitoylation: policing protein stability and traffic. *Nat. Rev. Mol. Cell Biol.* 8 74–84. 10.1038/nrm2084 17183362

[B31] Ling L. L. Schneider T. Peoples A. J. Spoering A. L. Engels I. Conlon B. P. (2015). A new antibiotic kills pathogens without detectable resistance. *Nature* 517 455–459. 10.1038/nature14098 25561178 PMC7414797

[B32] Lister P. D. Wolter D. J. Hanson N. D. (2009). Antibacterial-resistant *Pseudomonas aeruginosa*: clinical impact and complex regulation of chromosomally encoded resistance mechanisms. *Clin. Microbiol. Rev.* 22 582–610. 10.1128/CMR.00040-09 19822890 PMC2772362

[B33] Matias V. R. Al-Amoudi A. Dubochet J. Beveridge T. J. (2003). Cryo-transmission electron microscopy of frozen-hydrated sections of *Escherichia coli* and *Pseudomonas aeruginosa*. *J. Bacteriol.* 185 6112–6118. 10.1128/JB.185.20.6112-6118.2003 14526023 PMC225031

[B34] McCoy A. J. Grosse-Kunstleve R. W. Adams P. D. Winn M. D. Storoni L. C. Read R. J. (2007). Phaser crystallographic software. *J. Appl. Crystallogr.* 40 658–674. 10.1107/S0021889807021206 19461840 PMC2483472

[B35] Meroueh S. O. Bencze K. Z. Hesek D. Lee M. Fisher J. F. Stemmler T. L. (2006). Three-dimensional structure of the bacterial cell wall peptidoglycan. *Proc. Natl. Acad. Sci. U.S.A.* 103 4404–4409. 10.1073/pnas.0510182103 16537437 PMC1450184

[B36] Mima T. Sekiya H. Mizushima T. Kuroda T. Tsuchiya T. (2005). Gene cloning and properties of the RND-type multidrug eﬄux pumps MexPQ-OpmE and MexMN-OprM from *Pseudomonas aeruginosa*. *Microbiol. Immunol.* 49 999–1002. 10.1111/j.1348-0421.2005.tb03696.x 16301811

[B37] Miroux B. Walker J. E. (1996). Over-production of proteins in *Escherichia coli*: mutant hosts that allow synthesis of some membrane proteins and globular proteins at high levels. *J. Mol. Biol.* 260 289–298. 10.1006/jmbi.1996.0399 8757792

[B38] Mitra K. Ubarretxena-Belandia I. Taguchi T. Warren G. Engelman D. M. (2004). Modulation of the bilayer thickness of exocytic pathway membranes by membrane proteins rather than cholesterol. *Proc. Natl. Acad. Sci. U.S.A.* 101 4083–4088. 10.1073/pnas.0307332101 15016920 PMC384699

[B39] Morita Y. Tomida J. Kawamura Y. (2012). MexXY multidrug eﬄux system of *Pseudomonas aeruginosa*. *Front. Microbiol.* 3:408 10.3389/fmicb.2012.00408 PMC3516279 23233851

[B40] Nakajima A. Sugimoto Y. Yoneyama H. Nakae T. (2000). Localization of the outer membrane subunit OprM of resistance-nodulation-cell division family multicomponent eﬄux pump in *Pseudomonas aeruginosa*. *J. Biol. Chem.* 275 30064–30068. 10.1074/jbc.M005742200 10889211

[B41] Nakajima A. Sugimoto Y. Yoneyama H. Nakae T. (2002). High-level fluoroquinolone resistance in *Pseudomonas aeruginosa* due to interplay of the MexAB-OprM eﬄux pump and the DNA gyrase mutation. *Microbiol. Immunol.* 46 391–395. 10.1111/j.1348-0421.2002.tb02711.x 12153116

[B42] Nakayama H. Kurokawa K. Lee B. L. (2012). Lipoproteins in bacteria: structures and biosynthetic pathways. *FEBS J.* 279 4247–4268. 10.1111/febs.12041 23094979

[B43] Nikaido H. (2009). Multidrug resistance in bacteria. *Annu. Rev. Biochem.* 78 119–146. 10.1146/annurev.biochem.78.082907.145923 19231985 PMC2839888

[B44] Nikaido H. Pages J. M. (2012). Broad-specificity eﬄux pumps and their role in multidrug resistance of Gram-negative bacteria. *FEMS Microbiol. Rev.* 36 340–363. 10.1111/j.1574-6976.2011.00290.x 21707670 PMC3546547

[B45] Olivares J. Bernardini A. Garcia-Leon G. Corona F. Sanchez B. M. Martinez J. L. (2013). The intrinsic resistome of bacterial pathogens. *Front. Microbiol.* 4:103 10.3389/fmicb.2013.00103 PMC3639378 23641241

[B46] Phan G. Benabdelhak H. Lascombe M. B. Benas P. Rety S. Picard M. (2010). Structural and dynamical insights into the opening mechanism of *P. aeruginosa* OprM channel. *Structure* 18 507–517. 10.1016/j.str.2010.01.018 20399187

[B47] Poole K. (2004). Eﬄux-mediated multiresistance in Gram-negative bacteria. *Clin. Microbiol. Infect.* 10 12–26. 10.1111/j.1469-0691.2004.00763.x 14706082

[B48] Resh M. D. (1999). Fatty acylation of proteins: new insights into membrane targeting of myristoylated and palmitoylated proteins. *Biochim. Biophys. Acta* 1451 1–16. 10.1016/S0167-4889(99)00075-0 10446384

[B49] Robert X. Gouet P. (2014). Deciphering key features in protein structures with the new ENDscript server. *Nucleic Acids Res.* 42 W320–W324. 10.1093/nar/gku316 24753421 PMC4086106

[B50] Schweizer H. P. (2003). Eﬄux as a mechanism of resistance to antimicrobials in *Pseudomonas aeruginosa* and related bacteria: unanswered questions. *Genet. Mol. Res.* 2 48–62. 12917802

[B51] Su C. C. Radhakrishnan A. Kumar N. Long F. Bolla J. R. Lei H. T. (2014). Crystal structure of the *Campylobacter jejuni* CmeC outer membrane channel. *Protein Sci.* 23 954–961. 10.1002/pro.2478 24753291 PMC4088979

[B52] Tooley J. G. Schaner Tooley C. E. (2014). New roles for old modifications: emerging roles of N-terminal post-translational modifications in development and disease. *Protein Sci.* 23 1641–1649. 10.1002/pro.2547 25209108 PMC4253806

[B53] Urzhumtseva L. Afonine P. V. Adams P. D. Urzhumtsev A. (2009). Crystallographic model quality at a glance. *Acta Crystallogr. D Biol. Crystallogr.* 65 297–300. 10.1107/S0907444908044296 19237753 PMC2651759

[B54] Walsh C. (2003). Where will new antibiotics come from? *Nat. Rev. Microbiol.* 1 65–70. 10.1038/nrmicro727 15040181

[B55] Yen M. R. Peabody C. R. Partovi S. M. Zhai Y. Tseng Y. H. Saier M. H. (2002). Protein-translocating outer membrane porins of Gram-negative bacteria. *Biochim. Biophys. Acta* 1562 6–31. 10.1016/S0005-2736(02)00359-0 11988218

