# Supplementary material for: New OprM structure highlighting the nature of the N-terminal anchor
Source: Front Microbiol. 2015 Jul 1;6:667. doi: 10.3389/fmicb.2015.00667 (PMC4486845; doi:10.3389/fmicb.2015.00667)

Laura Monlezun et al. " New OprM structure highlighting the nature of the N-terminal anchor
Supplemental figures

Figure S1. Sequence alignment of the six OMF proteins with known structures.
The numbering corresponds to the OprM sequence after cleavage of the addressing signal
The secondary structure of OprM is indicated at the top of the aligned sequences.

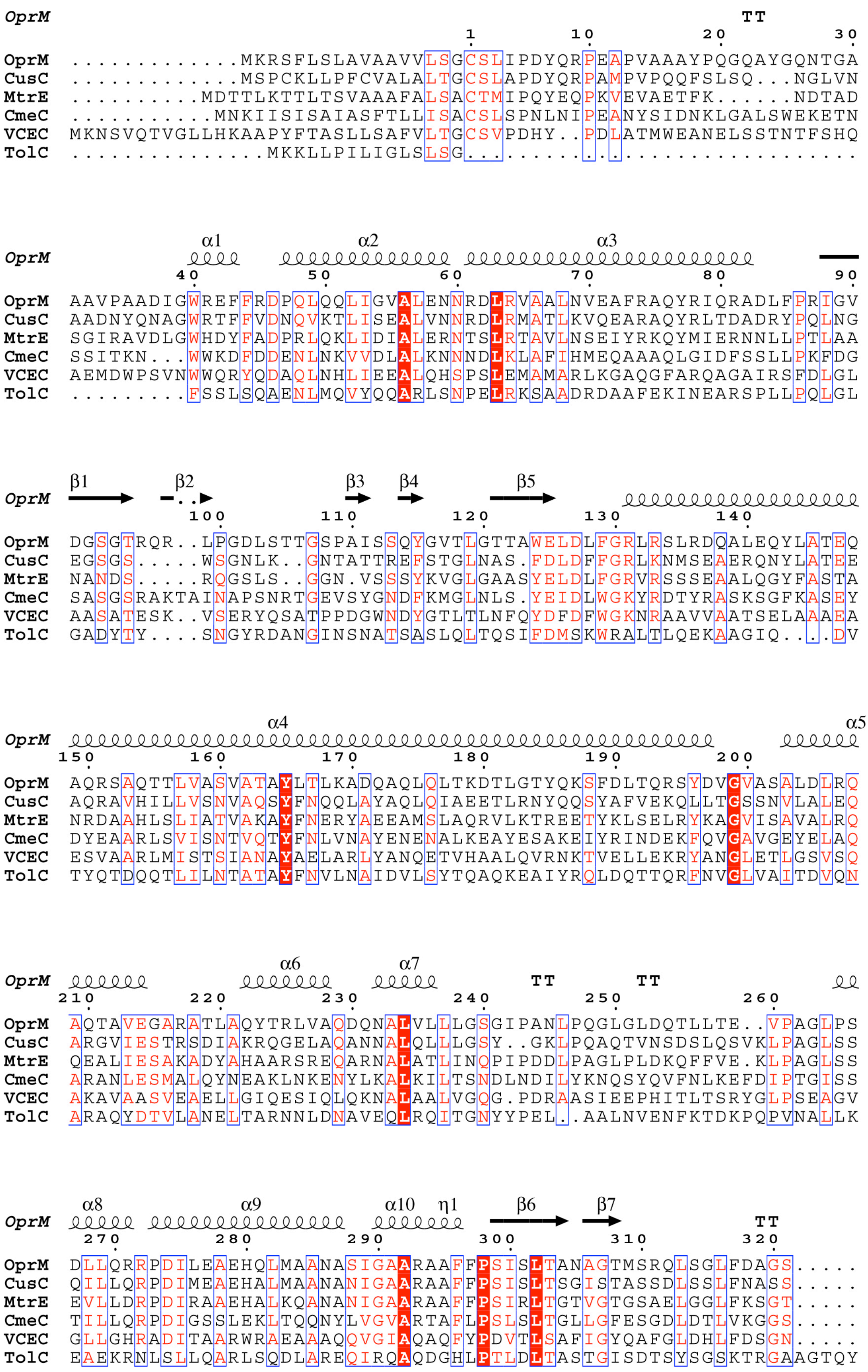

```
OprM        β8     β9                  α11                          α12
                  330       340       350       360       370
OprM  .....GSWLFQPSINLPIFTAGSLRASLDYAKIQKDINVAQYEKAIQTAFQEVADGLAAR
CusC  .....GMWNFIPKIEIPIFNAGRNQANLDIAEIRQQQSVVNYEQKIQNAFKEVADALALR
MtrE  .....GVWSFAPSITLPIFTWGTNKANLDVAKLRQQAQIVAYESAVQSAFQDVANALAAR
CmeC  .....KTWNIGGNFTLPIFHWGEIYQNVNLAKLNKDEAFVNYQNTLITAFGEIRYALVAR
VCEC  .....DAGAIGPAIYLPLFTGGRLEGQLTSAEARYQEAVAQYNGTLVQALHEIADVVTSS
TolC  DDSNMGQNKVGLSFSLPIYQGGMVNSQVKQAQYNFVGASEQLESAHRSVVQTVRSSFNNI

OprM                    α13                  α14   TTT    α15
        380       390       400       410       420       430
OprM  GTFTEQLQAQRDLVKASDEYYQLADKRYRTGVDNYLTLLDAQRSLFTAQQQLITDRLNQL
CusC  QSLNDQISAQQRYLASLQITLQRARALYQHGAVSYLEVLDAERSLFATRQTLLDLNYARQ
MtrE  EQLDKAYDALSKQSRASKEALRLVGLRYKHGVSGALDLLDAERSSYAAEGAALSAQLTRA
CmeC  KTIRLQYDNAQASEQSYKRIYEIAKERYDIGEMSLQDYLEARQNWLNAAVAFNNIKYSYA
VCEC  QALQARINKTEQAVQQAEQALHIATNRYQGGLATYLDVLVAEESLLNNQRALVNLQSRAF
TolC  NASISSINAYKQAVVSAQSSLDAMEAGYSVGTRTIVDVLDATTTLYNAKQELANARYNYL

      α16         η2
OprM
        440       450       460
OprM  TSEVNLYKALGGGWNQQTVTQQQTAKKEDPQA............................
CusC  VNEISLYTALGGG...............................................
MtrE  ENLADLYKALGGGLKRDTQTDK......................................
CmeC  NSIVDVIKAFGGGFEQSEDTSKNIKEESKNLDMSFRE.......................
VCEC  SLDLALIHALGGGFETTES.........................................
TolC  INQLNIKSALGTLNEQDLLALNNALSKPVSTNPENVAPQTPEQNAIADGYAPDSPAPVVQ

OprM

OprM  .................
CusC  .................
MtrE  .................
CmeC  .................
VCEC  .................
TolC  QTSARTTTSNGHNPFRN
```

Figure S2. Comparison of the two OprM packings: C2 (A) and $P2_12_12_1$ (B).
The unit cell is colored in red, and the cell parameters are indicated.

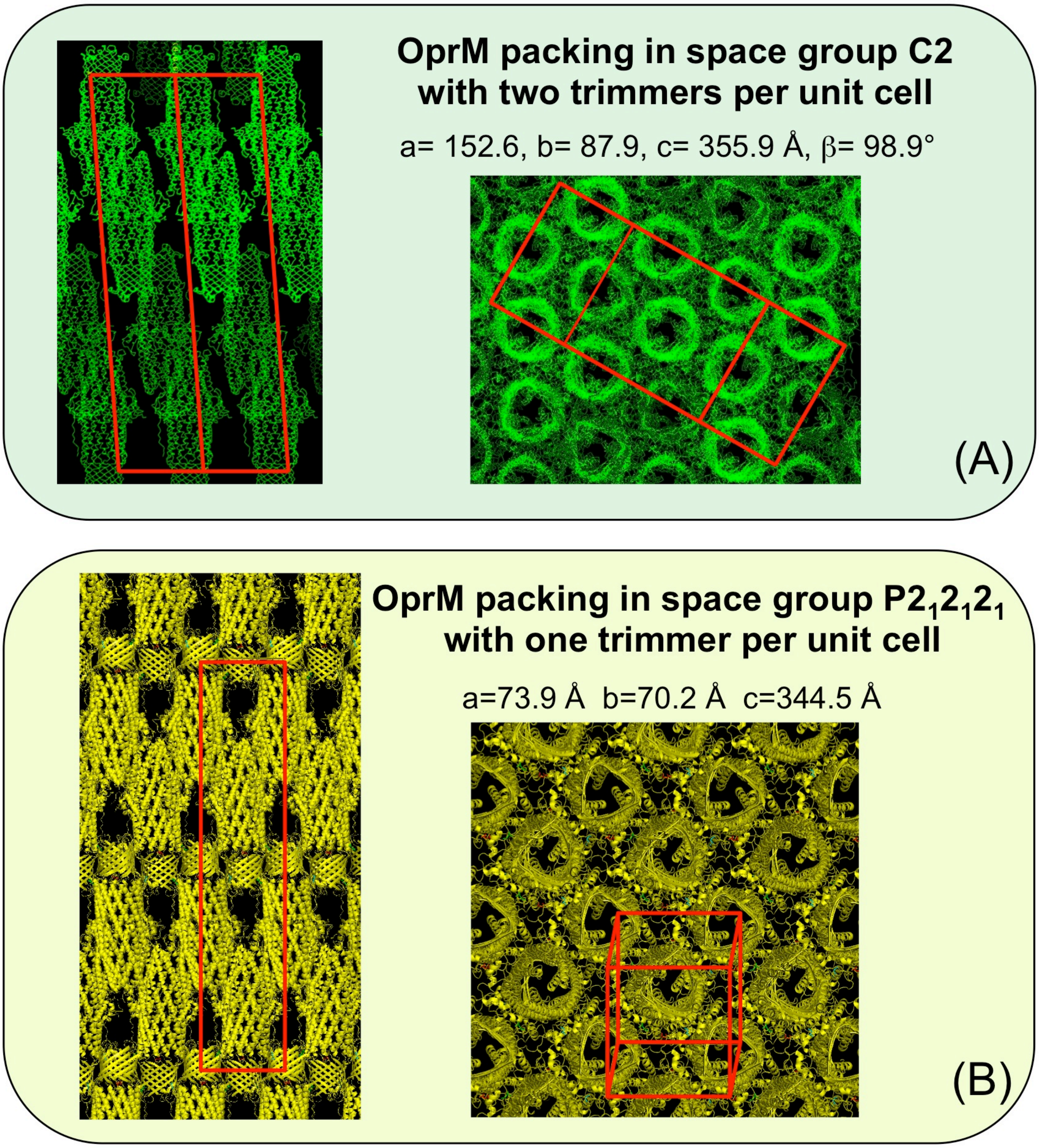

Figure S3. Quality assessment of the OprM structure refinement in the C2 space group by the polygon evaluation tool from Phenix

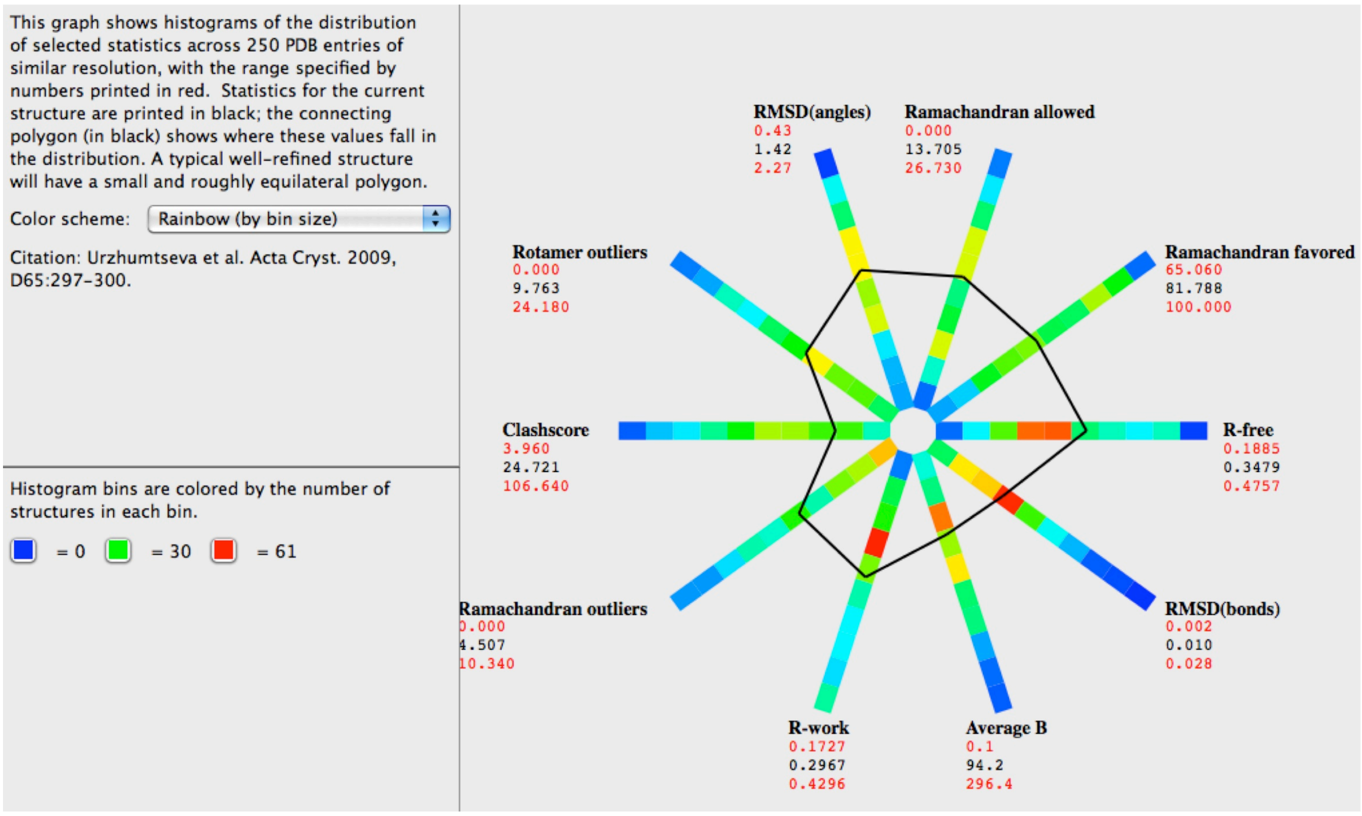

Supplement: Supplementary file 1 [file Image_1.PDF]
