# Supplementary material for: New OprM structure highlighting the nature of the N-terminal anchor
Source: Front Microbiol. 2015 Jul 1;6:667. doi: 10.3389/fmicb.2015.00667 (PMC4486845; doi:10.3389/fmicb.2015.00667)
Supplement: Supplementary file 2 [file Image_2.PDF]

Experimental material to be added to our answers to referee 1

Antibiograms of different *Pseudomonas aeruginosa* modified strains

The pME6001 plasmid used to transform *Pseudomonas* strains bears a gentamicin resistance gene

tested antibiotics are placed as follow (cefotaxime (CTX), piperacillin+tazobactam (TZP), piperacillin (PIP), cefepim (FEP), meropenem (MEM), ceftazidime (CAZ), ticarcillin + clavulanic acid (TCC), ticarcillin (TIC), aztreonam (ATM), tobramycin (TM), gentamicin (GM), imipenem (IPM), amikacin (AN), ciprofloxacin (CIP), colistin (CS), kanamycin (K))

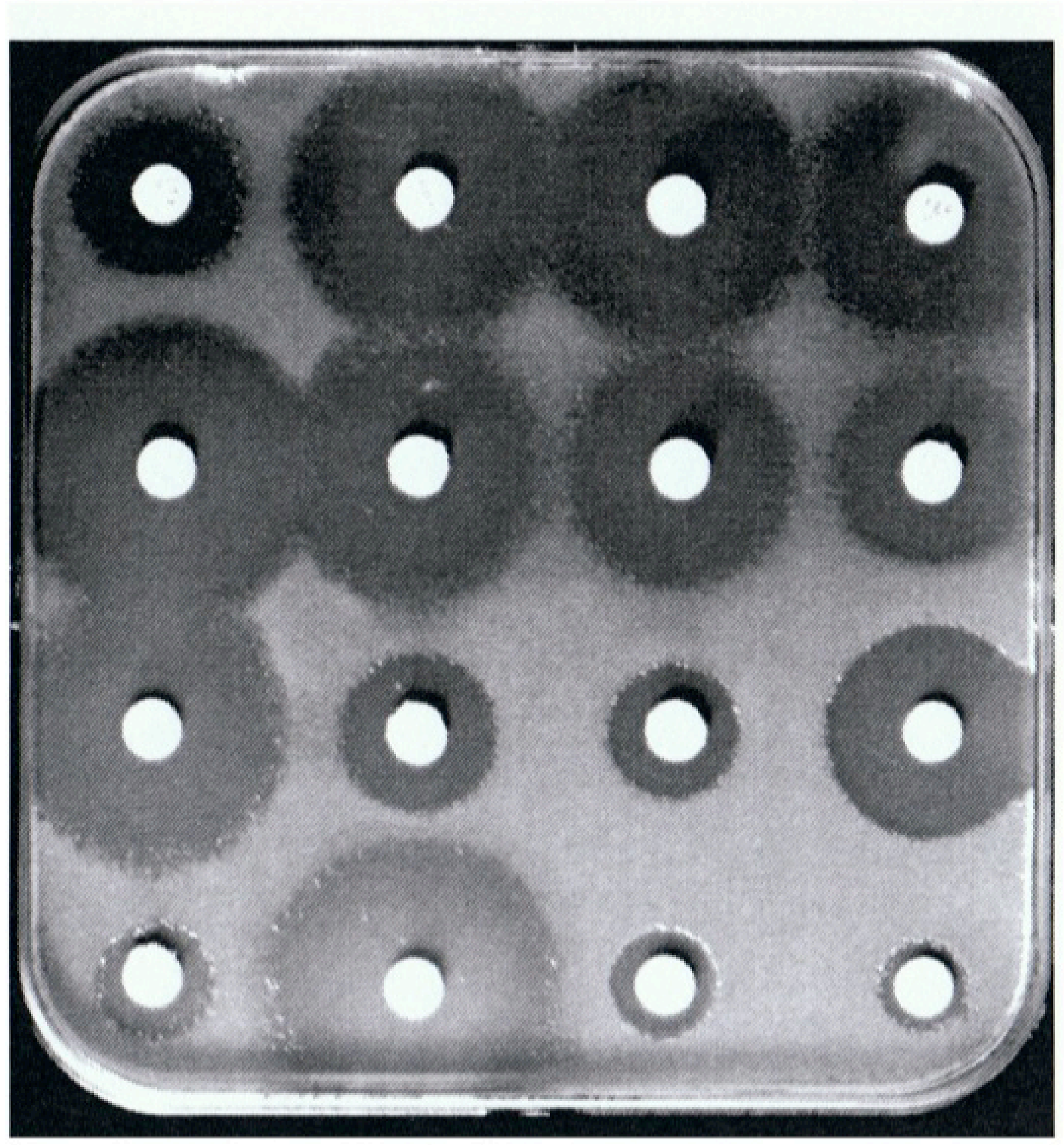

PAO1 WT

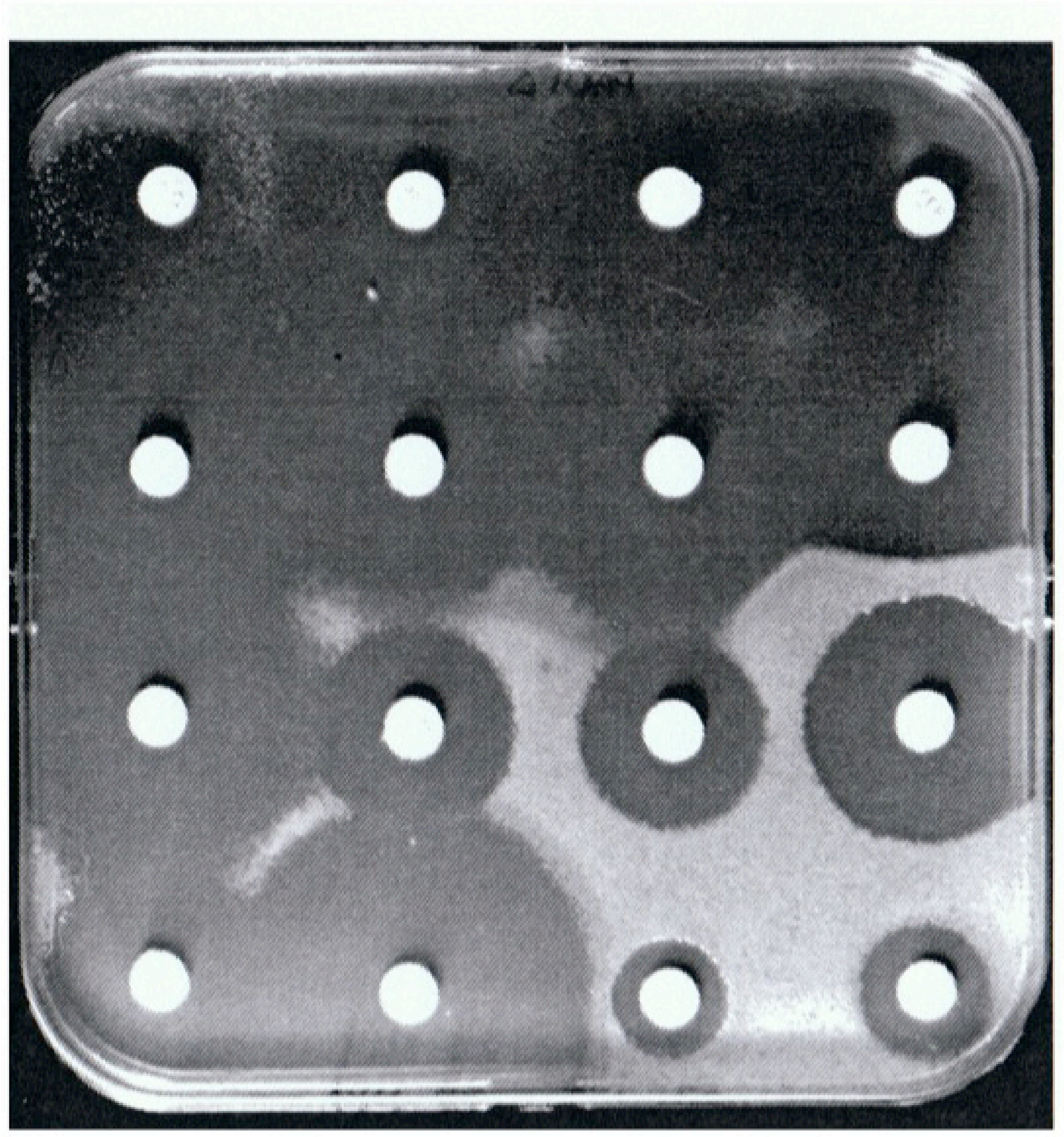

PAO1 ΔOprM

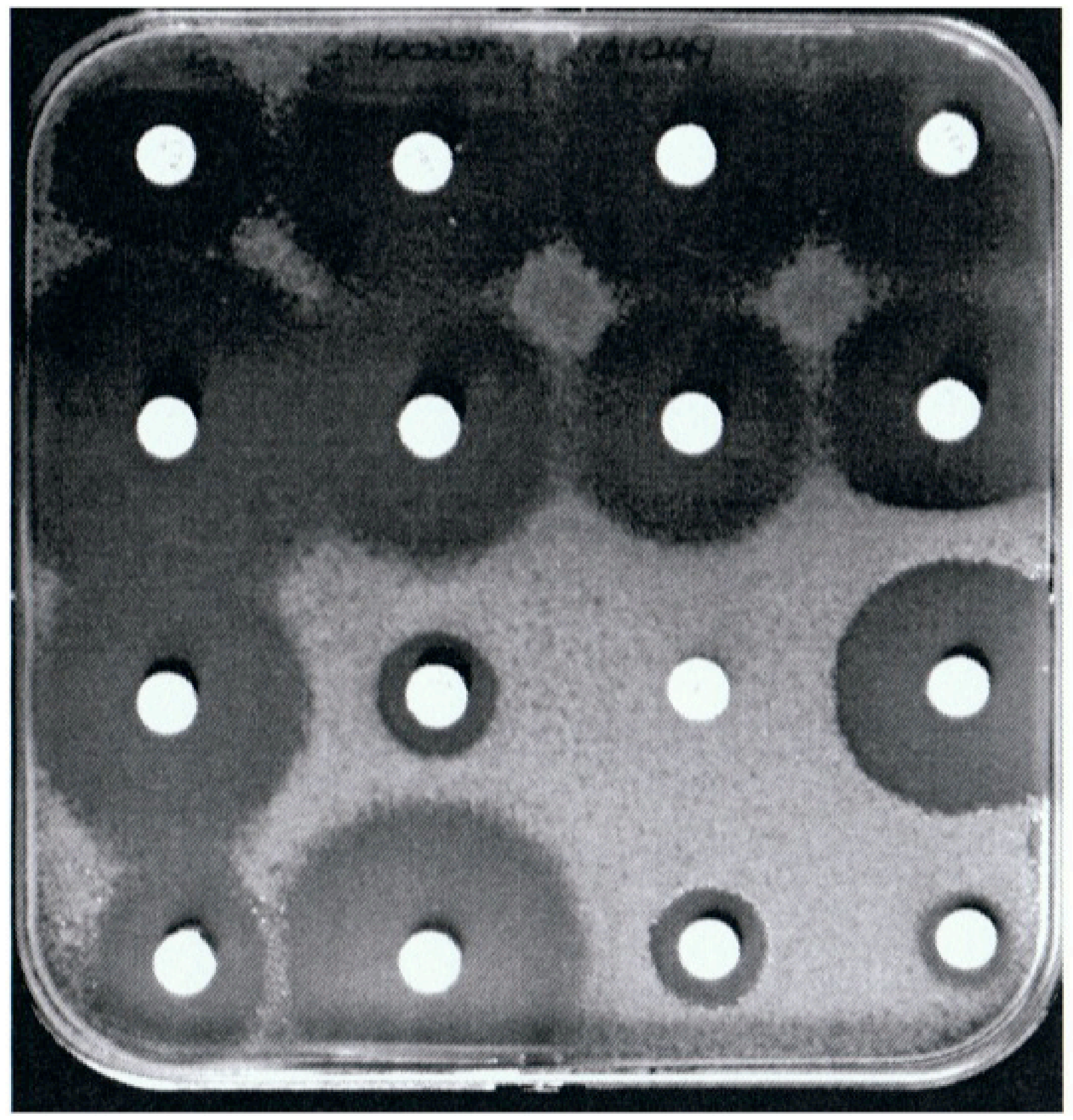

PAO1 ΔOprM + pME6001-OprMwt

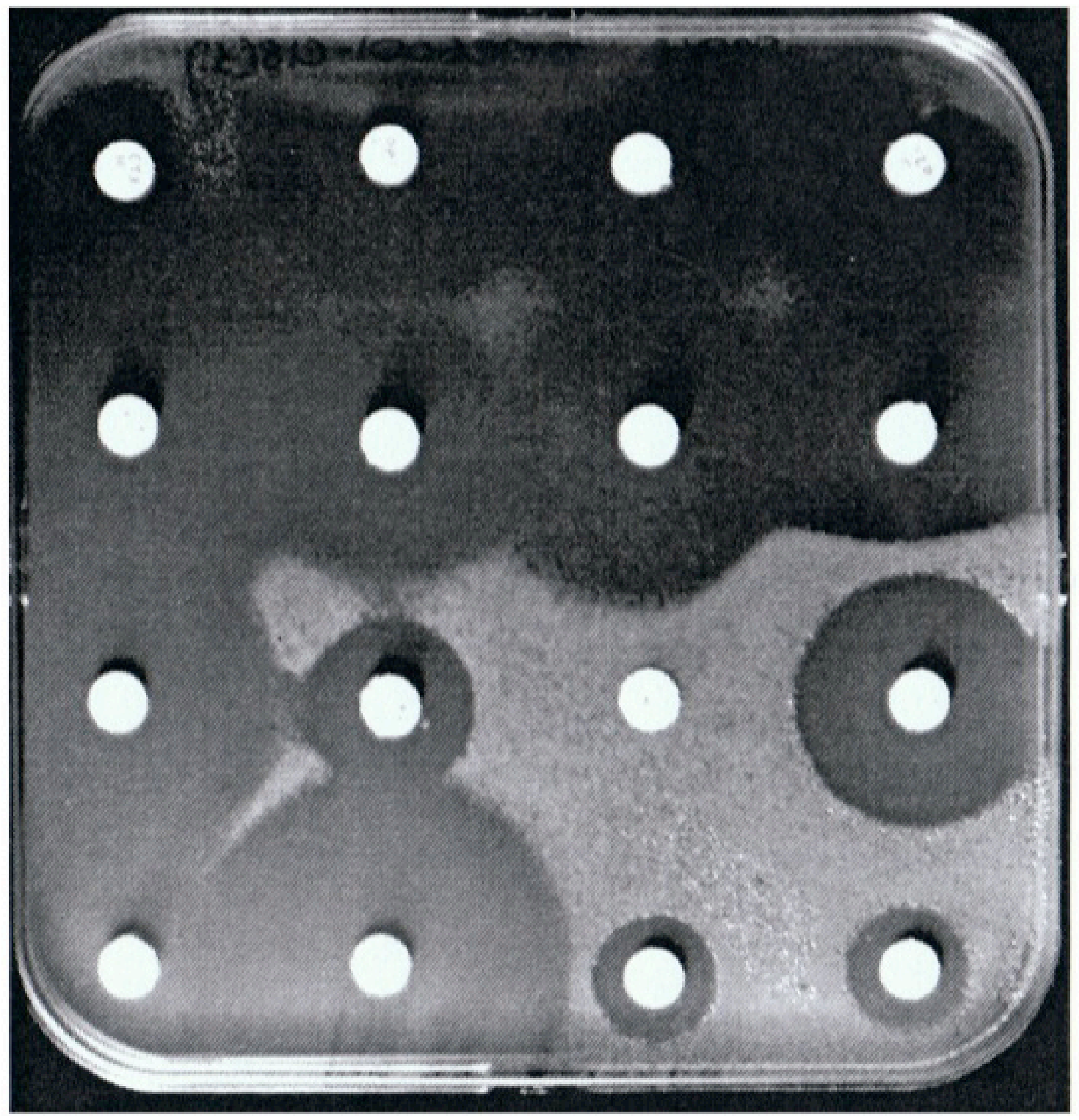

PAO1 ΔOprM + pME6001-spC18A-OprM
